# Utilization of the Emergency Department and Predicting Factors Associated With Its Use at the Saudi Ministry of Health General Hospitals

**DOI:** 10.5539/gjhs.v8n1p90

**Published:** 2015-04-15

**Authors:** Sundus O. Dawoud, Alaeddin Mohammad K. Ahmad, Omar Z. Alsharqi, Rajaa M. Al-Raddadi

**Affiliations:** 1 King Fahd Medical Research Center (KFMRC), Jeddah, Saudi Arabia; 2 Faculty of Economics and Administration, King Abdul-Aziz, University, Jeddah, Saudi Arabia; 3 King Abdul-Aziz University, Jeddah, Saudi Arabia

**Keywords:** Utilization of Emergency Department, general hospitals, MOH, Saudi Arabia

## Abstract

Overuse of emergency rooms (ER) is a public health problem. To investigate this issue, a cross-sectional survey was conducted at the ERs of King Abdul-Aziz Hospital, King Fahd Hospital, and Al-Thaghor Hospital in November 2013 with the aims of estimating emergency service utilization for non-urgent cases, identifying the predictors of ER utilization for non-urgent cases, and measuring patients’ knowledge of primary healthcare centers (PHCCs). Patients were interviewed using a structured questionnaire and the data were analyzed using the Statistical Package for the Social Sciences. We recruited 300 patients; males comprised 50.7% of the sample. A higher proportion of patients with non-urgent cases visited the ER three to four times a year (P=0.001). A higher proportion of patients without emergencies had not attempted to visit an outpatient clinic before the ER (P=0.003). Most patients without emergencies thought the ER was the first place to consult in case of illness. Most patients who visited the ER were single, < 15 years, and had lower incomes. Patients requested ER services for primary care-treatable conditions because of limited services and resources as well as limited working hours at PHCCs. Most patients (90.0%) were knowledgeable about PHCCs, with those of lower education being more knowledgeable. Patients reported long ER waiting times (≥ 3 hours), no organization (85.9%), and lack of medical staff. Overall, overuse of ER services is high at the Ministry of Health hospitals in Jeddah. The risk factors for ER overuse are age < 15 years, singlehood, and low incomes. Policy makers and health providers have a challenging task to control ER overuse. We recommend developing strategies to implement policies aimed at reducing non-urgent ER use as well as making healthcare services more available to the population.

## 1. Introduction

The unique characteristics of public health research are its focus on assessing, measuring, and monitoring the health of populations. However, traditional biomedical research deals with the study of diseases and treatments for individual patients (Lasker, 1997). When compared with other medical specialties, emergency medicine (EM) is well positioned to connect biomedical and public health approaches for preventing disease as well as injury and promoting health through population-based strategies targeted at the community (Clancy & Eisenberg, 1997).

Nowadays, access to the emergency department (ED) is available for two-thirds of patients admitted to hospitals in the United States (Clancy, 2007). In its strategically important position, at the border between the hospital and the surrounding community, the ED is actually the base of multiple systems of care. When all the systems are functioning, EM is available for all the patients 24 hours a day, seven days a week, irrespective of their ability to pay. EM offers care for both mental and physical health conditions and links patients with the most appropriate providers and care settings for presenting their conditions (Weissman, 1996).

The ED is also considered as a strong link between pre-hospital and in-hospital medical care, providing professional care for everyone at anytime (Gordon, 1999). More precisely, EM is the medical specialty with key missions, including evaluating, managing, treating, and preventing unexpected illness and injury (Rehmani, 2004). Therefore, several studies in Western societies examined factors that were associated with ED and their trend over the years (Meggs, Czaplijski, & Benson, 1999).

The ED also collects data to use for surveillance of infectious diseases such as sexually transmitted infections, tuberculosis, severe acute respiratory syndrome and environmental emergencies (e.g., heat waves, toxic spills) and forwards patient-level data to public health departments. However, research advancements and practical innovations are needed to enrich surveillance data by enabling ‘‘real-time’’ reporting of more cases and more complete data about individual cases (Weismann, 1996). In addition, the ED recognizes and attracts attention to major social problems that impact the health of the public including problems in food safety, homelessness, lack of health insurance or care coordination, child abuse, and interpersonal violence. EM also play a great role as a public health partner capable of monitoring and providing input regarding policies that affect public health along a number of dimensions (Weissman, 1996).

According to the Canadian national guidelines (Bakarman & Njaifan, 2014) which are followed by Saudi Ministry of Health (MOH) hospitals, patients must visit primary health care centers for examination, tests, and treatment, but if it is an emergency case converted to ED, and some emergency cases require ED directly. This study aims to estimate non-urgent ER visits at the Saudi MOH hospitals. It also aims to identify predictors of non-urgent ED utilization and assess patients’ knowledge of primary healthcare. There are many reasons which makes p0atients skip the primary healthcare system and use the ED e.g. lack of infrastructure of Saudi primary healthcare, also most of workers in the primary healthcare are non-Saudi which generated a weak communication with those staff.

## 2. Material Studied

### 2.1 Setting

This study was conducted at the ED of Saudi MOH hospitals (King Abdul-Aziz hospital, King Fahd hospital, and Al-Thaghor hospital), Jeddah, Kingdom of Saudi Arabia.

The ED of King Fahd hospital is an eight-room facility, with each room containing eleven beds: four for males and four for females. Two rooms are used for resuscitation, each of which has four beds. In addition, the ED of the hospital contains two clinics that run 24 hours daily. It also contains two triage rooms: very cold cases are not treated in one, while vital signs are measured and cases are triaged according to the Canadian classification in the other room.

King Abdul-Aziz hospital has a two-room ED. Each room, one for males and one for females, contains eleven beds. Two rooms are allotted for resuscitation, and each of the rooms has four beds. In addition, it has two clinics that run 24 hours daily. The ED also contains a triage room to measures vital signs and triage cases according to Canadian classifications.

The ED of Al-Thaghor hospital contains two rooms, each of which has nine beds. Of the two rooms, one is reserved for males and the other for females. The rooms for resuscitation each have two beds. In addition, the hospital ED contains one clinic that works 24 hours daily; however, it does not contain a triage room.

### 2.2 Design

This was a non-experimental, analytical cross-sectional study.

#### 2.2.1 Sample

We included all patients who presented to the ED of the above-mentioned hospitals in November 2013, irrespective of whether the cases was urgent or non-urgent.

#### 2.2.2 Sample Size

The calculated sample size for this study was 300 patients.

### 2.3 Data Collection

A structured questionnaire was used to collect data through interviews. The questionnaire included questions that assessed demographic data, factors that predicted overutilization of the emergency room (ER), and patients’ knowledge about primary health care services. The questionnaire identified many factors. This includes age, sex, nationality, job, educational background, marital status, health status, disease condition, reasons for visiting the emergency room, number of ED visits during the year, as well as knowledge about primary health care services and health care facilities. It also assessed the patients’ knowledge about when to consult an ER doctor and how to access the ED.

All cases were categorized as urgent and non-urgent according to the Canadian classification for ED attendance.

### 2.4 Validity and Reliability

The study instrument is a pretested and validated questionnaire (Porro et al., 2013). It was modified for use in this research, and it has been endorsed by experts.

### 2.5 Data Analysis

The data were analyzed using the Statistical Package for the Social Sciences (SSPS Inc., Chicago, IL, USA), version 20.0. Descriptive statistics were performed for all variables. The chi-square was used to assess the relationship between categorical variables, while the independent t-test was used to compare group means for continuous variables. Statistical significance was set at the 0.05 alpha level.

### 2.6 Ethical Consideration

Permission to conduct this study was granted by the ethics research committees of King Abdul-Aziz Hospital, King Fahd Hospital, and Al-Thaghor. Participants were informed that participation in this study was voluntary. Informed consent was obtained from all participants prior to their inclusion in this study. The consent form, which also explained the purpose of this study, was included in the questionnaire. All data were confidential and used solely for the purpose of this study.

## 3. Results

### 3.1 Estimation of Emergency Room Utilization

Three hundred patients, 100 from each of the three MOH, were included in this study. Males and females constituted approximately equal proportions of the sample (Table 1). Most patients were aged 16-23 years; patients >60 years comprised the lowest proportion of patients (7.3%). Over half of the patients had not completed at least high school and earned salaries between 3000-5000 SR; 20 patients had high salaries (> 15001 SR).

**Table 1:** Demographic characteristics of the sample

Demographics	Frequency	Percent
**Hospital**		
General King Fahd	100	33.3
King Abdul-Aziz	100	33.3
Al-Thaghor	100	33.3
**Gender**		
Male	152	50.7
Female	148	49.3
**Marital status**		
Married	138	46.0
Single	155	51.7
Divorced/Widow	7	2.3
**Age (years)**		
< 8	40	13.3
8 - 15	40	13.3
16 - 23	60	20.0
24 - 31	45	15.0
32 - 40	44	14.7
41 - 60	49	16.3
> 60	22	7.3
**Educational level**		
Less than high school	179	59.7
High school/Diploma	85	28.3
Bachelor/Post Graduate	36	12.0
**Income (SR)**		
3000 - 5000	156	52.0
5001 - 8000	67	22.3
8001 - 11000	39	13.0
11,001-15,000	18	6.0
> 15001	20	6.7

Of the 300 cases, 53.0% were non urgent. Al-Thaghor Hospital received a significantly high number of non-urgent ER cases as compared with the other two hospitals (Table 2).

**Table 2:** Relationship between emergency status and hospitals

Hospital	Emergency Status		P-value

	Non-urgent	Urgent	
King Fahad	34 (34.0)	66 (66.0)	
King Abdul-Aziz	53 (53.0)	47 (47.0)	<0.001*
Al-Thaghor	72 (72.0)	28 (28.0)

*Note.* Data are presented as frequency (percent) unless otherwise stated.

* Significant using a chi-square test at the 0.05 level.

As shown in Table 3, patients frequently visited the ER for non-urgent health problems. In particular, a significantly high proportion of patients visited the ER three to four times a year (68.5%) for non-urgent health issues (P=0.001). A significantly higher proportion of non-urgent cases had experienced symptoms of one to two day’s duration (P=0.001).

**Table 3:** Relationship between emergency status and patients’ experience at the emergency room

Variables	Emergency Status		Total (N=300)	P-value

	Non-urgent	Urgent		
Do you have a pre-file in the hospital?				
Yes	75 (50.0)	75 (50.0)	150 (100.0)	0.298
No	84 (56.0)	66 (44.0)	150 (100.0)	
How many times have you come to the emergency during the year?				
Frequently	9 (29.0)	22 (71.0)	31 (100.0)	
One to two months	6 (42.9)	8 (57.1)	14 (100.0)	
Three to four months	50 (68.5)	23 (31.5)	73 (100.0)	
Six months to one year	65 (57.5)	48 (42.5)	113 (100.0)	0.001*
Rarely	29 (42.0)	40 (58.0)	69 (100.0)	
Since when are you suffering from this problem?				
Suddenly	13 (33.3)	26 (66.7)	39 (100.0)	
One to two days	104 (62.3)	63 (37.7)	167 (100.0)	
One to two weeks	30 (50.0)	30 (50.0)	60 (100.0)	0.001*
Almost a month	6 (60.0)	4 (40.0)	10 (100.0)	
More than a month	6 (25.0)	18 (75.0)	24 (100.0)	

*Note.* Data are presented as frequency (percent) unless otherwise stated.

* Significant using a chi-square test at the 0.05 level.

A significantly higher proportion of patients without emergencies had not attempted to see a doctor at an outpatient clinic prior to visiting the ER (58.7% versus 41.3% for urgent cases). Of the 92 patients who had visited the ER, 37 (40.2%) without emergencies had attempted to see a doctor at an outpatient clinic as against 55 (59.8%) with emergency health issues who had attempted to visit an outpatient clinic prior to visiting the ER (P=0.003).

**Figure 1:**
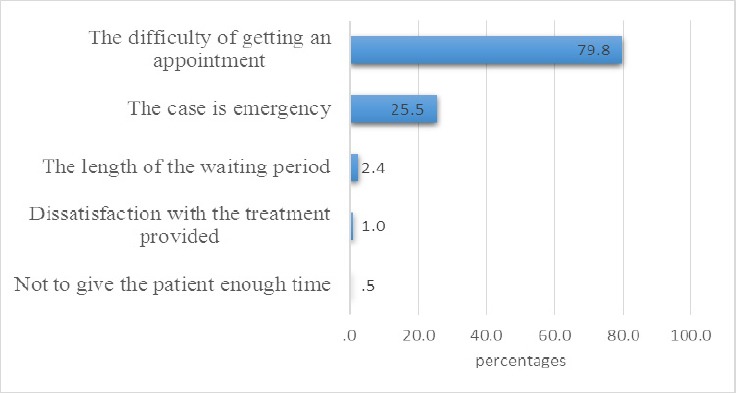
Reasons why patients had not seen a specialist doctor

A total of 208 patients had not attempted to see a specialist doctor before an ER visit. Difficulty in getting an appointment was cited as the most common reason why patients did not visit a specialist doctor prior to an ER visit. Other reasons are as shown in Figure 1.

We found that 119 patients (71.7%) with non-urgent health issues as against 47 (28.3%) with emergency health problems had difficulties in getting an appointment (P<0.001). Only three patients with non-emergencies (7.1%) versus 39 (92.9%) with emergencies reported not having difficulties getting an appointment. In total, 166 patients had difficulties getting an appointment at a specialized clinic.

A significantly higher proportion of patients without emergency conditions thought the ER was the first place to consult in case of illness (57.1% versus 42.9% for patients with emergencies; P = 0.020). Forty-eight patients with emergencies (57.8%) as against 35 without emergencies (42.2%) thought the ER was the first place to visit in case of illness. Overall, 217 patients (72.3%) consulted an ER doctor first in case of illness.

**Figure 2:**
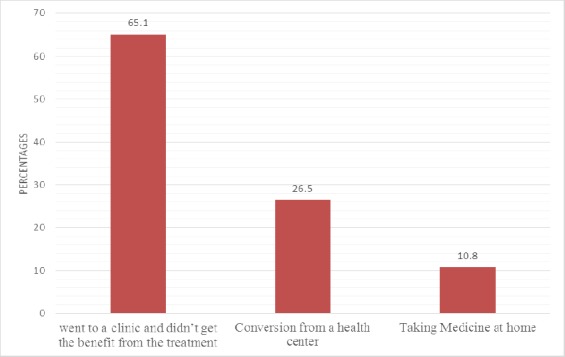
Patients options when they experienced symptoms

Approximately 65.1% of the 83 patients who went to a clinic reported that the treatment was unsatisfactory, and only 26.5% (eight being non-urgent cases) were referred from PHCCs to an ER (Figure 2).

Of the patients who consulted at the ER, 274 (91.3%) had never been denied services although 148 cases were not emergencies (Table 4). However, these results did not reach statistical significance.

**Table 4:** Action of emergency room staff toward urgent and non-urgent cases

Variables	Emergency Status		Total	P-value

	Non-urgent	Urgent		
Have you ever gone to the emergency department and the staff apologized that they could not receive you because your case was not an emergency?				
Yes	11 (42.3)	15 (57.7)	26 (100.0)	
No	148 (54.0)	126 (46.0)	274 (100.0)	0.253
Total	159 (53.0)	141 (47.0)	300 (100.0)	
Did they give you an alternative solution to your health problem?				
Yes	6 (35.3)	11 (64.7)	17 (100.00	
No	5 (55.6)	4 (44.4)	9 (100.0)	0.320
Total	11 (42.3)	15 (57.7)	26 (100.0)	

*Note.* Data are presented as frequency (percent) unless otherwise stated. *Significant using a chi-square test at the 0.05 level.

Sixty-eight patients (81.0%) who had emergent conditions as against 16 (19.0%) without emergencies reported having chronic health problems (P < 0.001). On the other hand, 143 patients (66.2%) without emergencies versus 73 (33.8%) with emergencies reported not having chronic problems.

**Figure 3:**
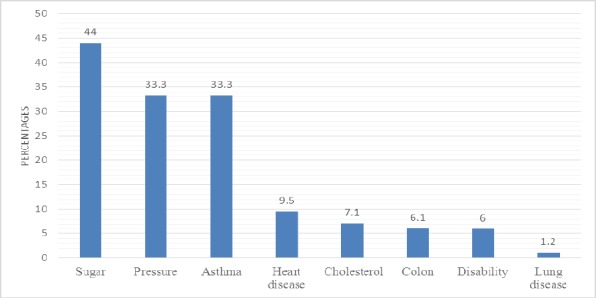
Common chronic health problems reported by the patients

Diabetes was the most common chronic health problem reported by the 84 patients, followed by high blood pressure and asthma (Figure 3).

### 3.2 Predictor of Emergency Room Utilization for Non-Emergent Conditions

A significant relationship existed between emergency status and the patient’s marital status (P < 0.001), age (P < 0.001), and income (P = 0.049). Most patients who visited the ER for non-urgent conditions were single, <15 years old, and had lower incomes (Table 5).

**Table 5:** Relationship between emergency status and demographic data

Variables	Emergency Status		Total (N=300)	P-value

	Non-urgent	Urgent		
**Marital status**				
Married	59 (42.8)	79 (57.2)	138 (100.0)	< 0.001*
Single	100 (64.5)	55 (35.5)	155 (100.0)	
Divorced	0 (0.0)	7 (100.0)	7 (100.0)	
**Age (years)**				
> 8	27 (67.5)	13 (32.5)	40 (100.0)	< 0.001*
8-15	28 (70.0)	12 (30.0)	40 (100.0)	
16-23	39 (65.0)	21 (35.0)	60 (100.0)	
24-31	22 (48.9)	23 (51.1)	45 (100.0)	
32-40	22 (50.0)	22 (50.0)	44 (100.0)	
41-60	17 (34.7)	32 (65.3)	49 (100.0)	
> 60	4 (18.2)	18 (81.8)	22 (100.0)	
**Income (SR)**				
3000-5000	80 (51.3)	76 (48.7)	156 (100.0)	0.049*
5001-8000	44 (65.7)	23 (34.3)	67 (100.0)	
8001-11000	14 (35.9)	25 (64.1)	39 (100.0)	
11001-15000	9 (50.0)	9 (50.0)	18 (100.0)	
> 15000	12 (60.0)	8 (40.0)	20 (100.0)	

*Note.* Data are presented as frequency (percent) unless otherwise stated.

* Significant using a chi-square test at the 0.05 level.

Of the 131 patients, over half (52.7%) with non-urgent conditions admitted that they got better quality services at the ER as compared to the treatment they received at clinics. However, 55.9% of 247 patients reported that they had the option to visit a clinic; 162 patients (54.3%) with non-urgent conditions had the option to visit a PHCC (Table 6).

**Table 6:** patients’ experiences that predicted emergency room utilization

Variables	Emergency Status		Total	P-value

	Non-urgent	Urgent		
**Do you get better services from the emergency room than doctor’s clinics?**				
Yes	69 (52.7)	62 (47.3)	131 (100.0)	0.920
No	90 (53.3)	79 (46.7)	169 (100.0)	
Total	159 (53.0)	141 (47.0)	300 (100.0)	
**Do you have a choice other than the emergency room to go when you become sick?**				
Yes	156 (53.2)	137 (46.8)	293 (100.0)	0.586
No	3 (42.9)	4 (57.1)	7 (100.0)	
Total	159 (53.0)	141 (47.0)	300 (100.0)	
**Telephone counseling**				
Yes	0 (0.0)	2 (100.0)	2 (100.0)	0.130
No	156 (53.2)	135 (46.4)	291 (100.0)	
Total	156 (53.2)	137 (46.8)	293 (100.0)	
**Primary health care centers**				
Yes	88 (54.3)	74 (45.7)	162 (100.0)	0.681
No	68 (51.9)	63 (48.1)	131 (100.0)	
Total	156 (53.2)	137 (46.8)	293 (100.0)	
**Visit outpatient clinics**				
Yes	138 (55.9)	109 (44.1)	247 (100.0)	0.037*
No	18 (39.1)	28 (60.9)	46 (100.0)	
Total	156 (53.2)	137 (46.8)	293 (100.0)	
**Go to the pharmacy**				
Yes	8 (50.0)	8 (50.0)	16 (100.0)	0.789
No	148 (53.4)	129 (46.6)	277 (100.0)	
Total	156 (53.2)	137 (46.8)	293 (100.0)	

*Note.* Data are presented as frequency (percent) unless otherwise stated.

* Significant using a chi-square test at the 0.05 level.

Approximately 56.0% of patients with non-urgent conditions admitted going to the ER although they knew that PHCCs could manage their cases. The main reasons cited by the patients were limited services and resources (n=162; 50.0%) and limited working hours (n=163; 63.8%) (Table 7).

**Table 7:** Relationship between emergency status and patients’ thoughts about primary healthcare centers

Variables	Emergency Status		Total	P-value

	Non-urgent	Urgent		
**If you knew that primary healthcare centers could deal with your case, would you go to the emergency room? If no, why?**				
Yes	150 (56.0)	118 (44.0)	268 (100.0)	0.003*
No	9 (28.1)	23 (71.9)	32 (100.0)	
Total	159 (53.0)	141 (47.0)	300 (100.0)	
**Limited services and resources**				
Yes	81 (50.0)	81 (50.0)	162 (100.0)	0.015*
No	69 (65.10)	37 (34.90)	7 (100.0)	
Total	150 (56.0)	118 (44.0)	268 (100.0)	
**Limited working hours**				
Yes	104 (63.8)	59 (36.2)	163 (100.0)	0.130
No	46 (43.8)	59 (56.2)	105 (100.0)	
Total	150 (56.0)	118 (44.0)	268 (100.0)	
**Lack of experience among medical staff**				
Yes	12 (44.4)	15 (55.6)	27 (100.0)	0.681
No	138 (57.3)	103 (42.7)	241 (100.0)	
Total	150 (56.0)	118 (44.0)	268 (100.0)	
**Dissatisfaction with the treatment provided**				
Yes	9 (47.4)	10 (52.6)	19 (100.0)	0.037*
No	141 (56.6)	108 (43.4)	249 (100.0)	
Total	150 (56.0)	118 (44.0)	293 (100.0)	
**Lack of effective diagnosis**				
Yes	5(29.4)	12 (70.6)	17 (100.0)	0.789
No	145 (57.8)	106 (42.2)	251 (100.0)	
Total	150 (56.0)	118 (44.0)	268 (100.0)	
**Mistrust of health centers**				
Yes	28 (42.4)	38 (57.6)	66 (100.0)	0.011*
No	122 (60.4)	80 (39.6)	202 (100.0)	
Total	150 (56.0)	118 (44.0)	268 (100.0)	
**Lack of knowledge of the health centers**				
Yes	9 (47.4)	10 (52.6)	19 (100.0)	0.433
No	141 (56.6)	108 (43.4)	249 (100.0)	
Total	150 (56.0)	118 (44.0)	268 (100.0)	

*Note.* Data are presented as frequency (percent) unless otherwise stated.

* Significant using a chi-square test at the 0.05 level.

**Figure 4:**
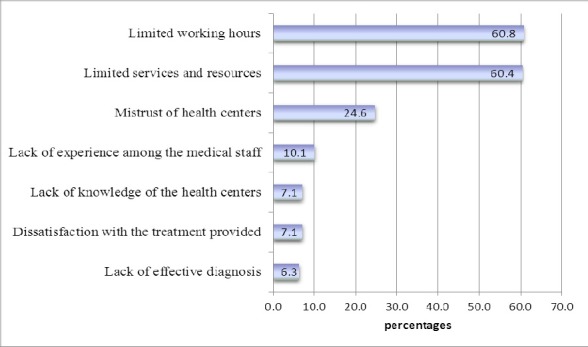
Reasons why patients visited the emergency room instead of primary healthcare centers

Although PHHCs could offer the same services, patients cited various reasons for not using their services (Figure 4).

**Figure 5:**
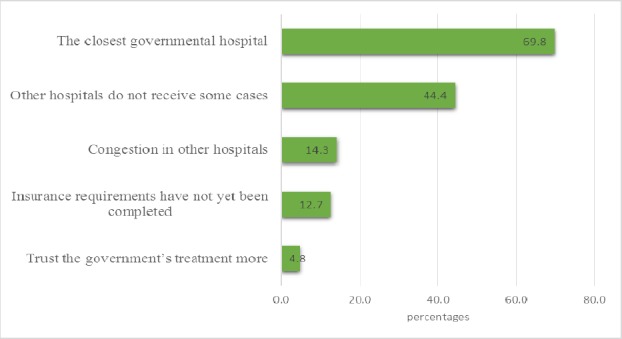
Reason why patients visit the emergency room despite having health insurance

Forty-two patients (66.7%) without emergent conditions versus 21 (33.3%) with emergency conditions had health insurance (P=0.014). In 36 (57.1%) of the 63 patients, health insurance was issued by King Fahd Armed Forces Hospital; 18 (28.6%) had private insurance, and nine (14.3%) had insurance issued by National Guard Hospital (P< 0.014). Of the 237 patients who did not have insurance, those with non-urgent conditions comprised 49.4% of the sample. The reasons why patients go to the ER despite having health insurance are shown in Figure 5.

### 3.3 Patients’ Knowledge about Primary Healthcare

Ninety percent of the patients were knowledgeable about PHCCs although some were not knowledgeable about the services offered by these centers (Table 8). The mean score of the patients (n=270) was 73.6 (SD, 32.8).

**Table 8:** Assessment of the patients’ knowledge about primary healthcare centers

Measuring Knowledge	Frequency	Percent
**Do you know what primary healthcare centers are?**		
Yes	270	90.0
No	30	10.0
Total	300	100.0
**Is there a primary healthcare center in the neighborhood where you live?**		
Yes	258	95.6
No	12	4.4
Total	270	100.0
**Do you know how to access its services?**		
Yes	215	79.6
No	55	20.4
Total	270	100.0
**Do you have a file at a primary healthcare center?**		
Yes	223	82.6
No	47	17.4
Total	270	100.0
**Public clinics**		
Yes	186	68.9
No	84	31.1
Total	270	100.0
**Clinics chronic diseases**		
Yes	185	68.5
No	85	31.5
Total	270	100.0
**Clinics healthy child**		
Yes	185	68.5
No	85	31.5
Total	270	100.0
**Clinics bandaging**		
Yes	185	68.5
No	85	31.5
Total	270	100.0
**Do you know whether the primary healthcare center has an emergency department?**		
Yes	181	67.0
No	89	33.0
Total	270	100.0
**Do you know their working hours?**		
Yes	225	83.3
No	45	16.7
Total	270	100.0
**Do you know the services provided by the centers?**		
Yes	185	68.5
No	85	31.5
Total	270	100.0
**Do you know that the primary healthcare centers can treat most of the cases that come to the emergency room?**		
Yes	87	29.0
No	213	71.0
Total	300	100.0

The patients’ mean knowledge score regarding PHCCs was 73.62 (SD 32.8). Further analysis showed that there was no significant relationship between patients’ knowledge of services offered by PHCCs and emergency status.

### 3.4 Impact of overcrowding on the emergency room

Table 9 shows that 44.9% of 136 patients who had urgent conditions reported that they did not get treatment as they expected. In addition, patients reportedly suffered long waiting times at the ER (up to three hours or more).

**Table 9:** Impact of overcrowding on the emergency room

Variables	Emergency Status		Total	P-value

	Non-urgent	Urgent		
**Did you get treatment in the emergency room as you had expected?**				
Yes	84 (51.2)	80 (48.8)	164 (100.0)	0.497
No	75 (55.1)	61 (44.9)	136 (100.0)	
Total	159 (59.4)	141 (52.6)	300 (100.0)	
**Did you suffer from long waiting times at the emergency room?**				
Yes	109 (57.4)	81 (42.6)	190 (100.0)	0.046*
No	50 (45.5)	60 (54.5)	110 (100.0)	
Total	159 (53.0)	141 (47.0)	300 (100.0)	
**For how many hours did you have to wait?**				
For an hour	56 (59.6)	38 (40.4)	94 (100.0)	0.039*
Two hours	43 (63.2)	25 (36.8)	68 (100.0)	
≥ Three hours	10 (35.7)	18 (64.3)	28 (100.0)	
Total	109 (57.4)	81 (42.6)	190 (100.0)
**Do you know that congestion of the emergency room may affect your health, treatment, and other patients?**				
Yes	147 (52.7)	132 (47.3)	279 (100.0)	0.693
No	12 (57.1)	9 (42.9)	21 (100.0)	
Total	159 (53.0)	141 (47.0)	300(100.0)	

*Note.* Data are presented as frequency (percent) unless otherwise stated.

* Significant using a chi-square test at the 0.05 level.

**Figure 6:**
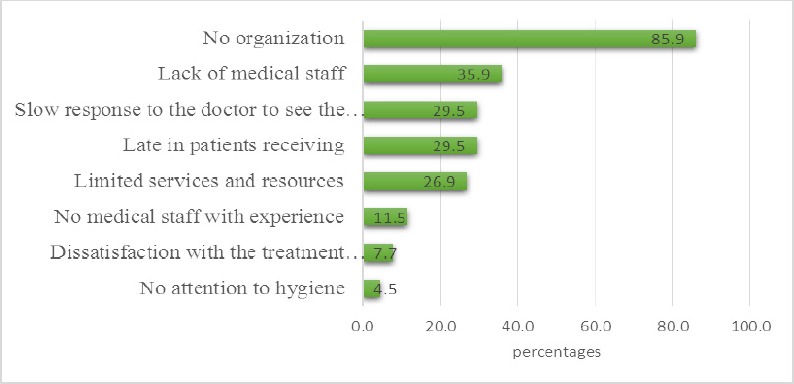
Problems Encountered by Patients at the Emergency Room

Besides long waiting times at the ER, 156 patients complained of no organization (85.9%), followed by lack of medical staff (35.9%: Figure 6).

Forty-six patients with non-urgent conditions reported having ever left the ER without receiving treatment versus 32 (41.0%) with urgent conditions (p=0.219). The main reason, as reported by 93.6% of the 78 patients, was because of overcrowding; 14.1% left because of the absence of a doctor.

A significantly higher proportion of patients with non-urgent conditions (n=151; 60.6%) visited the ED clinic as compared with patients with urgent problems (n=98; 39.4%; P<0.001). Of the 51 patients who visited the ER, eight patients (15.7%) had non-urgent conditions. Educational levels were significantly associated with patients’ knowledge about PHCCs, with those of lower educational level being more knowledgeable than those with a higher level (P = 0.007; Table 10).

**Table 10:** Relationship between knowledge of primary healthcare centers and educational level

Educational Level	Knowledge		Total (N=270)	P-value

	Poor (Less than 50%)	Acceptable (More than 50%)		
Less than high school	40 (24.1)	126 (75.9%)	166 (100.0)	
High school/Diploma	32 (42.1)	44 (57.9%)	76 (100.0)	
Bachelor/Post Graduate	12 (42.9)	16 (57.1%)	28 (100.0)	0.007*

*Note.* Data are presented as frequency (percent) unless otherwise stated.

* Significant using a chi-square test at the 0.05 level.

## 4. Discussion

Analysis of our data showed that the emergency services at the Saudi MOH hospitals were over-utilized for non-emergent cases. In addition, the frequency of non-urgent cases was significantly higher at Al-Thaghor Hospital as compared with the other two government hospitals. The percentage of non-urgent ER visits in this study is higher than that in other studies conducted abroad (Kellerman, 1994; Guttman, Zimmerman, & Nelson 2003; Northington, Brice, & Zou, 2005; National Center for Health Statistics, 2008; Carret, Fassa & Domingues, 2009; Durand, Gentile, & Devictor, 2011), where the authors reported that <10% of all ER visits were non-urgent. It is plausible that the high percentage of non-urgent ER visits in this study is due to ignorance, on the part of patients, of what constitutes an emergency case and lack of a hospital policy to discourage non-urgent visits. It is also possible that the hospital administration is afraid of being sued by patients for refusal to provide healthcare services, which may consequently tarnish the reputation of the hospital. Access to ER services was least for patients who consulted at King Fahd Hospital probably because the hospital has two triage rooms, one of which declines cold cases.

In this study, factors such as singlehood, younger age (less than 15 years), and lower incomes were significantly associated with non-urgent ER visits as compared with urgent cases. Patients of the younger age group were more likely to seek ER care for non-urgent conditions probably due to parental worries and its influence. Further, it is possible that the number of single persons is high because of the high proportion of young patients aged less than 15 years. Although there is limited evidence, one report (Uscher-Pines et al., 2013) suggests that younger age affects patients’ decisions to seek care in the ER for non-urgent conditions. Regarding income, people with low incomes are more likely to seek ER care at a Ministry of Health hospital because of the possibility of having free care and medications.

Convenience, which refers to the ease with which a patient can seek care, has been reported to be an important factor in driving non-urgent ER use. As depicted by the results of this study, patients with non-urgent conditions did not seek specialized services because of lack of convenience, which included difficulties in getting an appointment and long waiting times. In practice, it takes about a month to get an appointment at a MOH hospital, and it is difficult for patients with low incomes to go to private clinics. These further drive patients to seek care for non-urgent problems. Other studies (Sarver, Cydulka, & Baker, 2002; Harris Interactive, 2006) have also reported the role of convenience factors in driving non-urgent ER use. In one study (Redstone et al., 2008), it was demonstrated that 60% of non-urgent ER patients judged that ER care was more convenient than that provided at their PHCC.

A patient decides to seek care in an ER by consciously or unconsciously taking several factors into consideration. These may include prior visit to a doctor’s clinic and the patient’s experience of new-onset symptoms or a flare-up of a chronic condition that is not immediately debilitating, such as symptoms related to diabetes, asthma, or signs of stroke. The patient then has the choice of going to the ER or another location that can provide the same level of care or not seeking care. The results of this study showed that over half of the patients with non-urgent conditions decided to seek ER care. The most common reasons that drove patients to visit the ER care for non-urgent health problems were limited services, resources, and working hours as well as a lack of effective diagnosis at PHCCs. Although the organization of primary care services in Saudi Arabia has improved over the last decades, as confirmed by the reasonable number of staff in most PHCCs, studies point to several obstacles, including staff turnover (Al-Khaldi et al., 2002) and shortage of resources (Al-Khaldi & Al-Sharif, 2002). These obstacles discourage patients from using PHCCs. As an example, the lack of X-ray equipment or a technician at a PHCC would require that a physician transfers the patient to an ED, which would not only be time-consuming to the patient, but also more costly. Furthermore, PHCCs usually open during the day and only a few stay open till midnight, but never for 24 hours.

A significantly higher proportion of patients without emergencies had health insurance. About 66.7% of patients without emergencies versus 33.3% with emergencies had health insurance. Of the 63 patients with insurance, 57.1% had insurance by King Fahd Armed Forces Hospital, 28.6% had private insurance, and 14.3% had insurance by National Guard Hospital. Although other factors may have precluded patients with health insurance from consulting at other hospitals, reasons such as proximity and congestion at other governmental hospitals were most commonly cited by patients, and those who had private insurance reported insurance requirements have not yet been completed and some they would not pay the co-payment. Besides, the data suggest that ER staff do not discourage non-urgent ER visits, as shown by the high percentage of non-urgent cases that were received at the EDs of the hospitals. Findings from other studies suggest that cost sharing decreases use of appropriate and inappropriate health services (Newhouse, 1996), essential medications (Tambly, Laprise, & Hanley, 2001; Roblin, Platt, & Goodman, 2005), and preventive services (Redstone et al., 2008). These effects may occasionally cause worse health outcomes compared with health plans that involve lower out-of-pocket demands. Conversely, some studies (Blustein, 1995; Selby, Fireman, & Swain, 1996; Magid, Koepsell, & Every, 1997; Hsu, Price, & Brand, 2006) show that patients decrease elective services when ER care is subject to cost sharing, and this has not been associated with adverse outcomes. However, these observations may not apply to our context, especially when services are offered free of charge in hospitals such as King Fahd, King Abdul-Aziz, and Al-Thaghor hospitals. Moreover, in a developing country such as Saudi Arabia, there are still challenges in the health care insurance sector and many patients with lower incomes would rather seek care from the ER.

Emergency room users constitute a diverse population. While some users might visit the ER due to a habit or preference, others might opt for ER care due to lack of information regarding other options. In the current study, most patients were aware about PHCCs although they would visit the ER for primary care-treatable conditions because, as alluded to earlier, PHCCs had limited services, resources, and working hours and patients did not trust these centers. Patient dissatisfaction in clinics was also an important factor, as 65.1% of 83 patients who consulted at clinics did not find the treatment beneficial.

In particular, a significantly high proportion of patients (68.5%) in this study visited the ER three to four times a year for non-urgent conditions. Further, a significantly higher proportion of non-urgent cases had experienced symptoms of one to two days duration prior to visiting the ER. This typically causes overcrowding in ERs and consequently leads to sub-optimal care of patients with urgent conditions. Furthermore, crowding at the ER can lead to poor patient selection at the triage and the inadequate management of patients who present with acuity conditions. In fact, 44.9% of 136 patients who had urgent conditions in this study reported that not getting the treatment they expected. The patients’ reports of no organization at the ER, lack of medical staff, slow response of the doctor to see patients, competence and attitudes of other care providers, and long waiting times of up to three hours, which exceed the acceptable waiting limit set by Canadian guidelines, time spent at the ER have been previously reported by other authors (Pilpel, 1996; Goldwag et al., 2002) as factors that determined patient dissatisfaction with the ED.

Finally, patients’ educational levels affect their knowledge of PHCCs, with those of lower education being more knowledgeable than those with a higher education level. This may be because persons with lower education levels, owing to their lower incomes, would seek care at PHCCs because services are free of charge or very affordable.

## 5. Conclusion and Recommendation

Based on the findings of this study, the following conclusions are drawn:

The emergency services at the Ministry of Health hospitals in Jeddah were over-utilized, especially at Al-Thaghor hospital, which received a significantly high proportion of non-urgent cases. Singlehood, younger age, and lower incomes were significantly associated with non-urgent ER use. A significantly high proportion of patients visited the ER three to four times a year and at least six times in one year for non-urgent cases. A significantly higher proportion of patients with non-urgent conditions had experienced symptoms of one to two days duration. A considerable proportion of patients without emergencies had not attempted to see a doctor at an outpatient clinic prior to visiting the ER. In general, most patients would deter from consulting a specialist doctor before seeking emergency care in most cases because of difficulties in getting an appointment at a specialized clinic. A significantly higher proportion of patients without emergencies thought the ER was the first place to consult when they felt symptoms. Compared with patients with non-urgent conditions, a significant proportion of patients with urgent conditions had chronic problems. Although most patients were knowledgeable about PHCCs and the alternatives to seek care, a significant proportion would seek emergency care for non-urgent problems mainly due to negative perceptions about PHCCs and for convenience. A significant proportion of patients who had insurance had non-urgent conditions. Proximity and congestion at other governmental hospitals were the main reasons that drove them to consult emergency services. A significantly proportion of patients with urgent conditions admitted to the ED clinic as they should be admitted to ED room. Non-urgent ER visits resulted in congestion and consequently long waiting times of up to 3 hours. Patients’ educational levels were significantly associated with their knowledge of PHCCs, with those of lower education being more knowledgeable than those with a higher education.

The results of our analysis prompt us to make these recommendations:

Implement policies aimed at reducing non-urgent use of ERs such as not receive non-urgent cases and direct them to outpatient clinics or PHCC. Re-structure health care delivery systems to provide greater access to primary care and provide more attention to psychosocial aspects of patient care in clinical settings. Develop primary care services in organizations that will assume responsibility for health status, access and coordination of services for individuals and communities in ways that extend beyond their contact with the healthcare system and the provision of walk-in services. Strengthen primary care and promote the integration of service levels requires changing the Canada Health Act, which in fact limits coverage of services to those supplied by hospitals and physicians. Offices with multiple family doctors have to be more accessible. In addition, family doctors’ offices have to be close to the ER so that non-urgent cases can be easily referred to and addressed. Policy makers and healthcare providers should develop a health insurance policy that is commensurate with the expectation of the general population. Educate patients about emergency service use and improve their attitudes toward other health care choices. Introduce designated “fast track” units at Ministry of Health hospitals to entail the expeditious management of low acuity patients as well as the introduction of “fast track” improved waiting time for minor injuries without delaying the care of those with more serious injury. Limit boarding of patients in EDs by expanding hospital capacity. This will help in reducing the number of patients admitted at the ED and hence reduce overcrowding. Implement other strategies to address issues related to overcrowding in the various Ministry of Health hospitals, such as establish emergency centres for 24 hour across neighborhoods. Develop an information campaign to emphasize why and when attendance to the ER is inappropriate, and the negative impacts of this for the communication.

## References

[ref1] Al-Khaldi Y. M, Al-Sharif AI (2002). Availability Of Resources Of Diabetic Care In Primary Health Care Settings in Aseer region, Saudi Arabia. Saudi Medical Journal.

[ref2] Al-Khaldi Y. M, Al-Sharif A. I, Al-Jamal M. N, Kisha A. H (2002). Difficulties Faced When Conducting Primary Health Care Programs in Rural Areas. Saudi Medical Journal.

[ref3] Bakarman M, Njaifan N (2014). Assessment of Non-emergency Cases Attending Emergency Department at King Fahad General Hospital, Jeddah;Pattern and Outcomes. Life Science Journal.

[ref4] Blustein J (1995). Medicare Coverage, Supplemental Insurance, And The Use of Mammography by Older Women. The New England Journal of Medicine.

[ref5] Carret M. L, Fassa A. C, Domingues M. R (2009). Inappropriate Use of Emergency Services: A Systematic Review of Prevalence And Associated Factors. Cadernos Saude Publica.

[ref6] Clancy C (2007). Emergency Departments in Crisis: Implications For Quality And Safety. American Journal of Medical Quality.

[ref7] Clancy C, Eisenberg J (1997). Emergency Medicine in Population-Based Systems of Care. Emergency Medicine Clinics of North America.

[ref8] Durand A. C, Gentile S, Devictor B (2011). ED Patients: How Non urgent Are They? Systematic Review of The Emergency Medicine Literature. American Journal of Emergency Medicine.

[ref9] Goldwag R, Berg A, Yuval D, Benbassat J (2002). Predictors of Patient Dissatisfaction With Emergency Care. Israel Medical Association Journal.

[ref10] Gordon J (1999). The Hospital Emergency Department As A Social Welfare Institution. An Emergency Medicine Clinics of North America.

[ref11] Harris Interactive, Emergency Department Utilization in California: Survey of Consumer Data and Physician Data

[ref12] Hsu J, Price M, Brand R (2006). Cost-Sharing For Emergency Care and Unfavorable Clinical Events: Findings from the Safety and Financial Ramifications of ED Copayments Study. Health Service Research.

[ref13] Kellerman A. L (1994). Non urgent Emergency Department Visits: Meeting an Unmet Need. JAMA.

[ref14] Magid D. J, Koepsell T. D, Every N. R (1997). Absence of Association Between Insurance Copayments And Delays in Seeking Emergency Care Among Patients With Myocardial Infarction. New England Journal of Medicine.

[ref15] Meggs W, Czaplijski T, Benson N (1999). Trends in Emergency Department Utilization, 1988-1997. Academic Emergency Medicine.

[ref16] Miro O, Antonio M. T, Jiminez S, De Dios A, Sanchez M, Borras A, Milla J (1999). Decreased health care quality associated with emergency department overcrowding. Europe Journal of emergency medicine.

[ref17] Nadel V (1993). Emergency departments: unevenly affected by growth and change in patient use. US General Accounting Office: Report to the Chairman Subcommittee on health for families and the uninsured, (1993) committee on finance, us senate.

[ref18] Newhouse JP (1996). Free for All? Lessons From the RAND Health Insurance Experiment.

[ref19] Northington W. E, Brice J. H, Zou B (2005). Use of Emergency Department by Non Urgent Patients. American Journal of Emergency Medicine.

[ref20] Pilpel D (1996). Hospitalized Patients' Satisfaction with Caregivers' Conduct and Physical Surroundings. Journal of General Internal Medicine.

[ref21] Porro F, Monzani V, Folli C (2013). Reasons For Inappropriate Attendance of The Emergency Room In A Large Metropolitan Hospital. European Journal of Internal Medicine.

[ref22] Redstone P, Vancura J. L, Barry D, Kutner J. S (2008). Non Urgent Use of The Emergency Department. The Journal of Ambulatory Care Management.

[ref23] Rehmani R (2004). Emergency Medicine: A Relatively New Specialty (Editorial). Journal of Pakistan Medical Association.

[ref24] Roblin D. W, Platt R, Goodman M. J (2005). Effect of Increased Cost-Sharing on Oral Hypoglycemic Use in Five Managed Care Organizations: How Much Is Too Much?. Med Care.

[ref25] Sarver J. H, Cydulka R. K, Baker D. W (2002). Usual Source of Care And Non Urgent Emergency Department Use. Academic Emergency Medicine.

[ref26] Selby J. V, Fireman B. H, Swain B. E (1996). Effect of A Copayment on Use of The Emergency Department in A Health Maintenance Organization. New England Journal of Medicine.

[ref27] Tamblyn R, Laprise R, Hanley J. A (2001). Adverse Events Associated With Prescription Drug Cost-Sharing Among Poor and Elderly Persons. JAMA.

[ref28] Uscher-Pines L, Pines J, Kellermann A, Gillen E, Mehrotra A (2013). Emergency Department Visits For Non Urgent Conditions: Systematic Literature Review. The American Journal of Managed Care.

[ref29] Weissman J (1996). Uncompensated Hospital Care: Will It Be There if We Need It?. JAMA.

